# *catena*-Poly[diaqua­(*cis*-cyclo­hexane-1,2-dicarboxyl­ato)cadmium]

**DOI:** 10.1107/S1600536811044187

**Published:** 2011-10-29

**Authors:** Xiao-Hong Zhu, Xiao-Chun Cheng

**Affiliations:** a Faculty of Life Science and Chemical Engineering, Huaiyin Institute of Technology, Huaian 223003, People’s Republic of China

## Abstract

In the title polymer, [Cd(C_8_H_10_O_4_)(H_2_O)_2_]_*n*_, the Cd^II^ cation is coordinated by five carboxyl­ate O atoms from three different cyclo­hexane-1,2-dicarboxyl­ate anions and two O atoms from two water mol­ecules, displaying a distorted CdO_7_ pentagonal–bipyramidal geometry. Each anion acts as a μ_3_-bridge, linking symmetry-related Cd^II^ ions into a layer parallel to (010). In the crystal, numerous O—H⋯O and C—H⋯O hydrogen bonds occur. The coordinated water mol­ecules and carboxyl­ate O atoms act as donors or acceptors in the formation of these hydrogen-bonding inter­actions.

## Related literature

For related structures, see: Thirumurugan *et al.* (2006 ▶). 
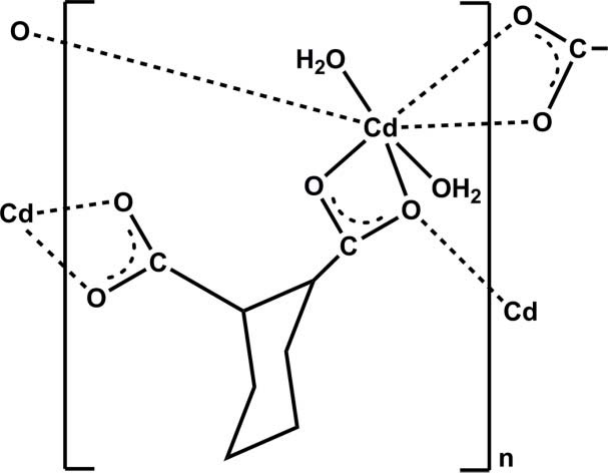


## Experimental

### 

#### Crystal data

[Cd(C_8_H_10_O_4_)(H_2_O)_2_]
*M* *_r_* = 318.59
Monoclinic, 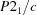

*a* = 6.0585 (9) Å
*b* = 23.544 (3) Å
*c* = 8.3308 (9) Å
β = 118.787 (8)°
*V* = 1041.5 (2) Å^3^
*Z* = 4
Mo *K*α radiation
μ = 2.10 mm^−1^
*T* = 293 K
0.20 × 0.20 × 0.18 mm

#### Data collection

Bruker SMART APEXII CCD diffractometer
Absorption correction: multi-scan (*SADABS*; Sheldrick, 1996 ▶) *T*_min_ = 0.678, *T*_max_ = 0.703
5908 measured reflections
2250 independent reflections
2214 reflections with *I* > 2σ(*I*)
*R*_int_ = 0.021

#### Refinement

*R*[*F* ^2^ > 2σ(*F* ^2^)] = 0.062
*wR*(*F* ^2^) = 0.132
*S* = 1.51
2250 reflections
136 parameters
H-atom parameters constrained
Δρ_max_ = 1.31 e Å^−3^
Δρ_min_ = −2.11 e Å^−3^

### 

Data collection: *APEX2* (Bruker, 2008 ▶); cell refinement: *SAINT* (Bruker, 2008 ▶); data reduction: *SAINT*; program(s) used to solve structure: *SHELXS97* (Sheldrick, 2008 ▶); program(s) used to refine structure: *SHELXL97* (Sheldrick, 2008 ▶); molecular graphics: *DIAMOND* (Brandenburg, 2000 ▶); software used to prepare material for publication: *SHELXTL* (Sheldrick, 2008 ▶).

## Supplementary Material

Crystal structure: contains datablock(s) I, global. DOI: 10.1107/S1600536811044187/pv2458sup1.cif

Structure factors: contains datablock(s) I. DOI: 10.1107/S1600536811044187/pv2458Isup2.hkl

Supplementary material file. DOI: 10.1107/S1600536811044187/pv2458Isup3.cdx

Additional supplementary materials:  crystallographic information; 3D view; checkCIF report

## Figures and Tables

**Table 1:** Hydrogen-bond geometry (Å, °)

*D*—H⋯*A*	*D*—H	H⋯*A*	*D*⋯*A*	*D*—H⋯*A*
O5—H11⋯O6^i^	0.85	2.01	2.828 (8)	164
O5—H12⋯O4^ii^	0.85	1.89	2.725 (8)	169
O6—H13⋯O3^iii^	0.85	1.85	2.694 (8)	175
O6—H14⋯O2^iv^	0.84	2.49	3.147 (8)	136
O6—H14⋯O4^iv^	0.84	2.57	3.016 (8)	115
C3—H3⋯O2	0.97	2.59	3.120 (10)	115
C6—H9⋯O4^v^	0.97	2.30	3.257 (10)	169

Symmetry codes: (i) 
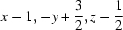
; (ii) 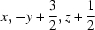; (iii) 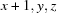; (iv) 
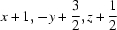
; (v) 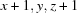.

**Fig. 1.**
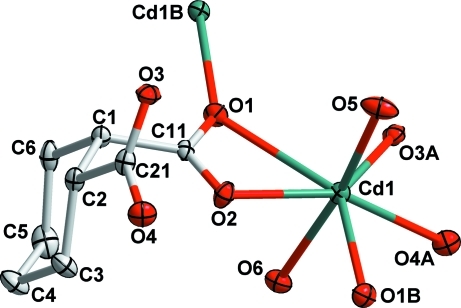
: The coordination environment of Cd ion in the title complex with the ellipsoids drawn at the 30% probability level. The hydrogen atoms are omitted for clarity. Symmetry code: A = 1 + x, 3/2 - y, 1/2 + z; B = x, 3/2 - y, 1/2 + z.

**Fig. 2.**
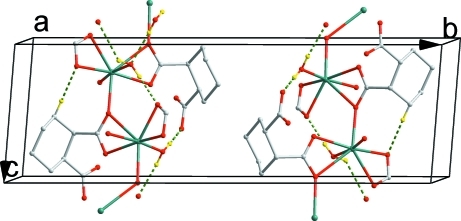
: The packing diagram of the title complex. Hydrogen bonds are shown in dashed lines.

## supplementary crystallographic information

### Comment

cyclohexane-1,2-dicarboxylic acid is often used as organic ligand to synthesize complexes for its variable conformation and coordination modes. Herein, we report the crystal structure of the title polymer. In contrast to the reported cadmium complex with cyclohexane-1,2-dicarboxylate (Thirumurugan *et al.*, 2006), the title complex crystallizes in a different space group, besides different carboxylate coordination modes and different crystal structure. The asymmetric unit of the title complex (Fig. 1) consists of a cadmium ion, a cyclohexane-1,2-dicarboxylate anion, and two coordinated water molecules. The Cd ion is coordinated by five carboxylate O atoms from three different cyclohexane-1,2-dicarboxylate anions, two O atoms from two coordinated water molecules, displaying a distorted CdO_7_ decahedral geometry. Each anion acts as a µ_3_-bridge, linking different cadmium ions to form a two-dimensional layer. In the crystal structure, there exist abundant O—H···O and C—H···O hydrogen bonds (Table 1, Fig. 1). Coordinated water molecules and carboxylate oxygen atoms act as donors or acceptors in the formation of these hydrogen bonding interactions.

### Experimental

Reaction mixture of cadmium perchlorate hexahydrate (49.4 mg, 0.1 mmol), cyclohexane-1,2-dicarboxylic acid (17.2 mg, 0.1 mmol) and potassium hydroxide (11.2 mg, 0.2 mmol) in 12 ml H_2_O was sealed in a 16 ml Teflon-lined stainless steel container and heated to 393 K for 3 days. After cooling to room temperature, colorless block crystals of the title complex were obtained.

### Refinement

The hydrogen atomsbonded to C atoms were located in geometrically idealized positions and constrained to ride on their parent atoms, with C—H = 0.97 or 0.98 Å and *U*_iso_(H) = 1.2*U*_eq_(C). The hydrogen atoms bonded to O5 and O6 were found from difference Fourier maps and fixed at those positions with [*U*_iso_(H) = 1.2*U*_eq_(O)]. The final difference map showed residual electron density in the close proximity of Cd-atom and was meaningless.

### Crystal data

**Table:**

[Cd(C_8_H_10_O_4_)(H_2_O)_2_]	*F*(000) = 632
*M**_r_* = 318.59	*D*_x_ = 2.032 Mg m^−^^3^
Monoclinic, *P*2_1_/*c*	Mo *K*α radiation, λ = 0.71073 Å
Hall symbol: -P 2ybc	Cell parameters from 3464 reflections
*a* = 6.0585 (9) Å	θ = 2.9–28.3°
*b* = 23.544 (3) Å	µ = 2.10 mm^−^^1^
*c* = 8.3308 (9) Å	*T* = 293 K
β = 118.787 (8)°	Block, colorless
*V* = 1041.5 (2) Å^3^	0.20 × 0.20 × 0.18 mm
*Z* = 4	

### Data collection

**Table:**

Bruker SMART APEXII CCD diffractometer	2250 independent reflections
Radiation source: fine-focus sealed tube	2214 reflections with *I* > 2σ(*I*)
graphite	*R*_int_ = 0.021
phi and ω scans	θ_max_ = 27.0°, θ_min_ = 1.7°
Absorption correction: multi-scan (*SADABS*; Sheldrick, 1996)	*h* = −7→7
*T*_min_ = 0.678, *T*_max_ = 0.703	*k* = −30→22
5908 measured reflections	*l* = −9→10

### Refinement

**Table:**

Refinement on *F*^2^	Primary atom site location: structure-invariant direct methods
Least-squares matrix: full	Secondary atom site location: difference Fourier map
*R*[*F*^2^ > 2σ(*F*^2^)] = 0.062	Hydrogen site location: inferred from neighbouring sites
*wR*(*F*^2^) = 0.132	H-atom parameters constrained
*S* = 1.51	*w* = 1/[σ^2^(*F*_o_^2^) + (0.0226*P*)^2^ + 8.9495*P*] where *P* = (*F*_o_^2^ + 2*F*_c_^2^)/3
2250 reflections	(Δ/σ)_max_ = 0.001
136 parameters	Δρ_max_ = 1.31 e Å^−^^3^
0 restraints	Δρ_min_ = −2.11 e Å^−^^3^

### Special details

**Table:**

Geometry. All e.s.d.'s (except the e.s.d. in the dihedral angle between two l.s. planes) are estimated using the full covariance matrix. The cell e.s.d.'s are taken into account individually in the estimation of e.s.d.'s in distances, angles and torsion angles; correlations between e.s.d.'s in cell parameters are only used when they are defined by crystal symmetry. An approximate (isotropic) treatment of cell e.s.d.'s is used for estimating e.s.d.'s involving l.s. planes.
Refinement. Refinement of *F*^2^ against ALL reflections. The weighted *R*-factor *wR* and goodness of fit *S* are based on *F*^2^, conventional *R*-factors *R* are based on *F*, with *F* set to zero for negative *F*^2^. The threshold expression of *F*^2^ > σ(*F*^2^) is used only for calculating *R*-factors(gt) *etc*. and is not relevant to the choice of reflections for refinement. *R*-factors based on *F*^2^ are statistically about twice as large as those based on *F*, and *R*- factors based on ALL data will be even larger.

### Fractional atomic coordinates and isotropic or equivalent isotropic displacement parameters (Å^2^)

**Table:**

	*x*	*y*	*z*	*U*_iso_*/*U*_eq_	
C1	0.2430 (14)	0.8769 (3)	0.3235 (9)	0.0215 (15)	
H1	0.1239	0.8731	0.3710	0.026*	
C2	0.0977 (14)	0.9021 (3)	0.1294 (9)	0.0217 (15)	
H2	0.0117	0.9360	0.1402	0.026*	
C3	0.2681 (16)	0.9226 (4)	0.0541 (11)	0.0306 (18)	
H4	0.1677	0.9430	−0.0595	0.037*	
H3	0.3423	0.8900	0.0268	0.037*	
C4	0.4788 (18)	0.9615 (4)	0.1884 (13)	0.040 (2)	
H6	0.5925	0.9702	0.1405	0.048*	
H5	0.4067	0.9969	0.2011	0.048*	
C5	0.6240 (16)	0.9336 (4)	0.3738 (12)	0.038 (2)	
H7	0.7527	0.9594	0.4574	0.046*	
H8	0.7071	0.8998	0.3625	0.046*	
C6	0.4523 (17)	0.9175 (3)	0.4512 (11)	0.0324 (19)	
H10	0.3772	0.9516	0.4694	0.039*	
H9	0.5506	0.8994	0.5694	0.039*	
C11	0.3489 (12)	0.8179 (3)	0.3255 (9)	0.0172 (13)	
C21	−0.1078 (14)	0.8624 (3)	0.0000 (10)	0.0233 (15)	
Cd1	0.52611 (10)	0.71211 (2)	0.27582 (7)	0.02114 (18)	
O1	0.4616 (10)	0.7907 (2)	0.4755 (7)	0.0292 (12)	
O2	0.3166 (11)	0.7961 (2)	0.1782 (7)	0.0286 (12)	
O3	−0.2409 (10)	0.8354 (2)	0.0540 (7)	0.0276 (12)	
O4	−0.1527 (11)	0.8576 (3)	−0.1638 (7)	0.0306 (13)	
O5	0.1442 (11)	0.6699 (3)	0.1849 (8)	0.0420 (16)	
H11	0.0396	0.6864	0.0873	0.050*	
H12	0.0655	0.6580	0.2390	0.050*	
O6	0.8811 (10)	0.7702 (2)	0.3509 (7)	0.0291 (12)	
H13	0.8529	0.7914	0.2610	0.035*	
H14	0.9968	0.7461	0.3832	0.035*	

### Atomic displacement parameters (Å^2^)

**Table:**

	*U*^11^	*U*^22^	*U*^33^	*U*^12^	*U*^13^	*U*^23^
C1	0.026 (4)	0.018 (4)	0.018 (3)	0.002 (3)	0.009 (3)	0.000 (3)
C2	0.024 (4)	0.019 (3)	0.016 (3)	0.003 (3)	0.005 (3)	0.000 (3)
C3	0.034 (4)	0.029 (4)	0.026 (4)	−0.009 (3)	0.012 (3)	0.003 (3)
C4	0.042 (5)	0.034 (5)	0.047 (5)	−0.014 (4)	0.023 (5)	0.002 (4)
C5	0.021 (4)	0.034 (5)	0.041 (5)	−0.001 (3)	0.001 (4)	0.000 (4)
C6	0.039 (5)	0.022 (4)	0.024 (4)	0.002 (3)	0.006 (4)	−0.005 (3)
C11	0.015 (3)	0.016 (3)	0.020 (3)	−0.001 (3)	0.007 (3)	−0.002 (3)
C21	0.020 (3)	0.022 (4)	0.019 (3)	0.004 (3)	0.003 (3)	−0.001 (3)
Cd1	0.0190 (3)	0.0229 (3)	0.0189 (3)	0.0022 (2)	0.0070 (2)	0.0012 (2)
O1	0.031 (3)	0.031 (3)	0.022 (3)	0.006 (2)	0.011 (2)	0.005 (2)
O2	0.031 (3)	0.030 (3)	0.021 (3)	0.006 (2)	0.010 (2)	−0.003 (2)
O3	0.026 (3)	0.030 (3)	0.030 (3)	−0.007 (2)	0.017 (2)	−0.003 (2)
O4	0.029 (3)	0.039 (3)	0.019 (3)	0.000 (3)	0.007 (2)	0.001 (2)
O5	0.030 (3)	0.064 (4)	0.027 (3)	−0.006 (3)	0.010 (3)	0.014 (3)
O6	0.023 (3)	0.034 (3)	0.028 (3)	0.000 (2)	0.010 (2)	0.007 (2)

### Geometric parameters (Å, °)

**Table:**

C1—C11	1.528 (9)	C11—O1	1.271 (9)
C1—C6	1.534 (11)	C21—O4	1.262 (9)
C1—C2	1.540 (10)	C21—O3	1.266 (9)
C1—H1	0.9800	C21—Cd1^i^	2.737 (7)
C2—C21	1.515 (10)	Cd1—O2	2.278 (5)
C2—C3	1.522 (11)	Cd1—O5	2.286 (6)
C2—H2	0.9800	Cd1—O3^ii^	2.337 (5)
C3—C4	1.531 (11)	Cd1—O1^iii^	2.340 (5)
C3—H4	0.9700	Cd1—O6	2.365 (5)
C3—H3	0.9700	Cd1—O4^ii^	2.407 (6)
C4—C5	1.512 (12)	Cd1—O1	2.639 (6)
C4—H6	0.9700	Cd1—C21^ii^	2.737 (7)
C4—H5	0.9700	O1—Cd1^iv^	2.340 (5)
C5—C6	1.512 (13)	O3—Cd1^i^	2.337 (5)
C5—H7	0.9700	O4—Cd1^i^	2.407 (6)
C5—H8	0.9700	O5—H11	0.8466
C6—H10	0.9700	O5—H12	0.8458
C6—H9	0.9700	O6—H13	0.8460
C11—O2	1.256 (9)	O6—H14	0.8404
			
C11—C1—C6	110.9 (6)	O3—C21—Cd1^i^	58.4 (4)
C11—C1—C2	112.8 (6)	C2—C21—Cd1^i^	178.1 (5)
C6—C1—C2	110.6 (6)	O2—Cd1—O5	87.6 (2)
C11—C1—H1	107.4	O2—Cd1—O3^ii^	137.40 (19)
C6—C1—H1	107.4	O5—Cd1—O3^ii^	98.8 (2)
C2—C1—H1	107.4	O2—Cd1—O1^iii^	82.2 (2)
C21—C2—C3	113.0 (6)	O5—Cd1—O1^iii^	90.7 (2)
C21—C2—C1	111.5 (6)	O3^ii^—Cd1—O1^iii^	139.2 (2)
C3—C2—C1	113.4 (6)	O2—Cd1—O6	82.6 (2)
C21—C2—H2	106.1	O5—Cd1—O6	170.2 (2)
C3—C2—H2	106.1	O3^ii^—Cd1—O6	88.65 (19)
C1—C2—H2	106.1	O1^iii^—Cd1—O6	87.78 (19)
C2—C3—C4	112.4 (7)	O2—Cd1—O4^ii^	157.3 (2)
C2—C3—H4	109.1	O5—Cd1—O4^ii^	111.1 (2)
C4—C3—H4	109.1	O3^ii^—Cd1—O4^ii^	54.90 (18)
C2—C3—H3	109.1	O1^iii^—Cd1—O4^ii^	84.63 (19)
C4—C3—H3	109.1	O6—Cd1—O4^ii^	78.4 (2)
H4—C3—H3	107.9	O2—Cd1—O1	52.46 (17)
C5—C4—C3	110.9 (7)	O5—Cd1—O1	94.6 (2)
C5—C4—H6	109.5	O3^ii^—Cd1—O1	84.97 (17)
C3—C4—H6	109.5	O1^iii^—Cd1—O1	133.97 (13)
C5—C4—H5	109.5	O6—Cd1—O1	79.61 (19)
C3—C4—H5	109.5	O4^ii^—Cd1—O1	134.15 (17)
H6—C4—H5	108.0	O2—Cd1—C21^ii^	158.3 (2)
C4—C5—C6	111.3 (7)	O5—Cd1—C21^ii^	107.8 (2)
C4—C5—H7	109.4	O3^ii^—Cd1—C21^ii^	27.5 (2)
C6—C5—H7	109.4	O1^iii^—Cd1—C21^ii^	112.0 (2)
C4—C5—H8	109.4	O6—Cd1—C21^ii^	81.7 (2)
C6—C5—H8	109.4	O4^ii^—Cd1—C21^ii^	27.4 (2)
H7—C5—H8	108.0	O1—Cd1—C21^ii^	109.6 (2)
C5—C6—C1	111.7 (7)	C11—O1—Cd1^iv^	143.0 (5)
C5—C6—H10	109.3	C11—O1—Cd1	84.6 (4)
C1—C6—H10	109.3	Cd1^iv^—O1—Cd1	130.9 (2)
C5—C6—H9	109.3	C11—O2—Cd1	102.0 (4)
C1—C6—H9	109.3	C21—O3—Cd1^i^	94.1 (4)
H10—C6—H9	107.9	C21—O4—Cd1^i^	91.0 (5)
O2—C11—O1	120.8 (6)	Cd1—O5—H11	106.8
O2—C11—C1	119.6 (6)	Cd1—O5—H12	135.0
O1—C11—C1	119.6 (6)	H11—O5—H12	108.1
O4—C21—O3	119.8 (7)	Cd1—O6—H13	109.7
O4—C21—C2	119.9 (7)	Cd1—O6—H14	101.8
O3—C21—C2	120.2 (7)	H13—O6—H14	117.6
O4—C21—Cd1^i^	61.6 (4)		
			
C11—C1—C2—C21	54.4 (8)	O3^ii^—Cd1—O1—C11	176.1 (4)
C6—C1—C2—C21	179.2 (6)	O1^iii^—Cd1—O1—C11	9.9 (6)
C11—C1—C2—C3	−74.6 (8)	O6—Cd1—O1—C11	86.6 (4)
C6—C1—C2—C3	50.2 (9)	O4^ii^—Cd1—O1—C11	149.0 (4)
C21—C2—C3—C4	−178.5 (7)	C21^ii^—Cd1—O1—C11	163.8 (4)
C1—C2—C3—C4	−50.3 (9)	O2—Cd1—O1—Cd1^iv^	166.6 (4)
C2—C3—C4—C5	53.1 (11)	O5—Cd1—O1—Cd1^iv^	83.2 (3)
C3—C4—C5—C6	−57.3 (11)	O3^ii^—Cd1—O1—Cd1^iv^	−15.2 (3)
C4—C5—C6—C1	58.5 (10)	O1^iii^—Cd1—O1—Cd1^iv^	178.65 (11)
C11—C1—C6—C5	71.9 (8)	O6—Cd1—O1—Cd1^iv^	−104.7 (3)
C2—C1—C6—C5	−54.0 (9)	O4^ii^—Cd1—O1—Cd1^iv^	−42.3 (4)
C6—C1—C11—O2	−123.1 (7)	C21^ii^—Cd1—O1—Cd1^iv^	−27.5 (4)
C2—C1—C11—O2	1.5 (10)	O1—C11—O2—Cd1	−4.1 (8)
C6—C1—C11—O1	59.1 (9)	C1—C11—O2—Cd1	178.1 (5)
C2—C1—C11—O1	−176.2 (6)	O5—Cd1—O2—C11	99.8 (5)
C3—C2—C21—O4	−12.9 (10)	O3^ii^—Cd1—O2—C11	−0.5 (6)
C1—C2—C21—O4	−142.0 (7)	O1^iii^—Cd1—O2—C11	−169.1 (5)
C3—C2—C21—O3	169.7 (7)	O6—Cd1—O2—C11	−80.4 (5)
C1—C2—C21—O3	40.5 (9)	O4^ii^—Cd1—O2—C11	−113.9 (6)
O2—C11—O1—Cd1^iv^	−162.3 (6)	O1—Cd1—O2—C11	2.1 (4)
C1—C11—O1—Cd1^iv^	15.5 (12)	C21^ii^—Cd1—O2—C11	−36.3 (9)
O2—C11—O1—Cd1	3.5 (7)	O4—C21—O3—Cd1^i^	4.1 (7)
C1—C11—O1—Cd1	−178.8 (6)	C2—C21—O3—Cd1^i^	−178.4 (6)
O2—Cd1—O1—C11	−2.1 (4)	O3—C21—O4—Cd1^i^	−4.0 (7)
O5—Cd1—O1—C11	−85.5 (4)	C2—C21—O4—Cd1^i^	178.6 (6)

Symmetry codes: (i) *x*−1, −*y*+3/2, *z*−1/2; (ii) *x*+1, −*y*+3/2, *z*+1/2; (iii) *x*, −*y*+3/2, *z*−1/2; (iv) *x*, −*y*+3/2, *z*+1/2.

### Hydrogen-bond geometry (Å, °)

**Table:**

*D*—H···*A*	*D*—H	H···*A*	*D*···*A*	*D*—H···*A*
O5—H11···O6^i^	0.85	2.01	2.828 (8)	164.
O5—H12···O4^iv^	0.85	1.89	2.725 (8)	169.
O6—H13···O3^v^	0.85	1.85	2.694 (8)	175.
O6—H14···O2^ii^	0.84	2.49	3.147 (8)	136.
O6—H14···O4^ii^	0.84	2.57	3.016 (8)	115.
C3—H3···O2	0.97	2.59	3.120 (10)	115.
C6—H9···O4^vi^	0.97	2.30	3.257 (10)	169.

Symmetry codes: (i) *x*−1, −*y*+3/2, *z*−1/2; (iv) *x*, −*y*+3/2, *z*+1/2; (v) *x*+1, *y*, *z*; (ii) *x*+1, −*y*+3/2, *z*+1/2; (vi) *x*+1, *y*, *z*+1.
